# The perspectives of recipients and their partners conceiving through oocyte donation on counselling and healthcare: A qualitative study

**DOI:** 10.1177/17455057251374891

**Published:** 2025-10-07

**Authors:** Kim van Bentem, Eileen Lashley, Amber Visser, Marloes Vermeulen, Moniek ter Kuile, Marie-Louise van der Hoorn

**Affiliations:** 1 Department of Obstetrics and Gynaecology, Leiden University Medical Center, the Netherlands; 2 Department of Obstetrics and Gynaecology, Erasmus Medical Center, Rotterdam, the Netherlands; 3 Department of Health, Medical and Neuropsychology, Institute of Psychology, University of Leiden, the Netherlands; 4 External Contacts Officer at Freya (Dutch Association for People with Fertility Problems), Amersfoort, the Netherlands; 5 Department of Psychiatry and Psychology, Leiden University Medical Center, the Netherlands

**Keywords:** oocyte donation, qualitative research, focus groups, patient perspectives, preconception counselling, pregnancy

## Abstract

**Background:**

Oocyte donation (OD), a treatment with increasing prevalence, introduces challenges in fertility and obstetric care, including pregnancy complications and psychosocial issues. As numerous healthcare providers encounter OD pregnancies, understanding the perspectives of involved stakeholders becomes crucial for improving OD healthcare management.

**Objectives:**

This study explores perspectives regarding counselling and healthcare of women and their partners conceiving through OD.

**Design:**

A qualitative case study design using a descriptive phenomenological approach.

**Methods:**

Three in-depth focus groups with 13 women and 5 partners, who had experienced OD pregnancy and/or delivery after treatment in either a Dutch or foreign centre, were conducted.

**Results:**

The findings show the significance of comprehensive counselling throughout the entire OD process. Preconception counselling was positively evaluated when various subjects were covered, not only the logistical part, but also emotional impact and ethical issues. However, the study revealed variations in type and quality of counselling provided, depending on healthcare provider. Participants often received contradictory information, and desired emotional and peer support. While psychosocial support was available before OD treatment in the Netherlands, it was often lacking in treatment abroad.

**Conclusion:**

To improve OD healthcare management, participants stated various recommendations. Mostly appointed was implementing a (inter)national guideline, which emphasizes the necessity for standardized and comprehensive counselling and healthcare for women and their partners undergoing OD treatment.

## Plain language summary

What women who conceive with donor eggs and their partners think about councelling and healthcare: A qualitative study

This study looks at what women and their partners think about the counselling and healthcare they received when having a baby through egg donation (also called oocyte donation, or OD). As more people use OD to have children, it is important for healthcare providers to understand their experiences, especially since OD pregnancies can come with extra medical and emotional challenges. Researchers organized three group discussions with 13 women and 5 partners who were pregnant or already had a child through OD, either in the Netherlands or abroad. The study found that good counselling is important throughout the entire OD process; from before pregnancy to after birth. Participants appreciated counselling that covered not just the medical steps, but also emotional and ethical aspects. However, the quality of counselling varied a lot. Some people got conflicting advice from different healthcare professionals, and many wanted more emotional support and chances to talk with others going through the same process. While mental support was often available in the Netherlands, it was missing in many international clinics. To improve OD healthcare, participants suggested creating clear guidelines. These would help ensure that everyone going through OD gets the same high-quality support and care.

## Introduction

The number of oocyte donation (OD) cycles is rising due to the possible consequence of postponement of pregnancy and increasing demand for fertility treatment, such as reception of the oocyte of the partner as part of shared lesbian motherhood.^1,2^ Furthermore, for certain medical conditions, such as Turner syndrome, OD represents the most feasible reproductive option.^3^ According to the most recent numbers of the European Society of Human Reproduction and Embryology (ESHRE), more than 8% of assisted reproductive treatment cycles are with donated oocytes.^1^ A variety of ethical and financial dilemmas are introduced by OD, as the procedure is strictly regulated or even forbidden in several countries within Europe,^4^ leading to expensive cross-border reproductive care.^5^

Counselling before and during OD pregnancy must be provided as OD pregnancies show a higher incidence of obstetrical complications compared to autologous artificially conceived pregnancies and naturally conceived pregnancies, including hypertensive complications, caesarean section and bleeding complications.^6–10^ In addition, OD may be accompanied with psychosocial stressors, such as mother–child relationship with the absence of a genetic bond.^11,12^ Preconception counselling will increase the alertness of patients and healthcare providers towards the risk profile of OD pregnancies,^13,14^ and the importance of an individualized surveillance and management strategy throughout pregnancy and delivery.^6^

To improve counselling and healthcare related to OD, it is important to identify the perspectives of involved stakeholders, including oocyte recipients and their partners. Current literature that addresses OD counselling is mainly focused on the psychosocial consequences of OD, such as motivation,^15–19^ disclosure towards offspring^20–27^ and the donor–recipient relationship.^28–30^ In research on mental health during OD parenthood,^31,32^ handling disclosure^20–22,24–26^ and the dilemma of choosing for non-anonymous or anonymous donation,^33,34^ the partner is also taken into account. Still, the experiences and perspectives on preconception counselling and OD pregnancy care remain unknown. However, it is highly relevant to identify the aspects that have been helpful, as well as those that require further development, in order to improve healthcare and ultimately improve outcomes for OD pregnancies.^6^ Therefore, in this exploratory study, we aim to elucidate the perspectives of both oocyte recipient and partner, regarding counselling before OD treatment and during pregnancy, including medical and emotional needs in fertility and obstetric care. The results of this study could lead to new insights in OD healthcare management and could be used in the development of a (inter)national guideline.

## Methods

### Study design

A qualitative case study design using a descriptive phenomenological approach was used to provide an elaborate description of lived experiences of the OD recipients and their partners, hereafter referred to as participants.^35,36^ Three in-depth focus groups were conducted, as the interactive nature allows to produce information that might not be gathered from an individual interview. Focus groups are valuable for stimulating discussion, obtaining a wider range of perspectives and supporting participants in memorizing and sharing their perspectives; therefore, sensitive and personal disclosures are more likely to arise compared to individual interviews.^37^ The study was conducted by the department of Obstetrics and Gynaecology in the Leiden University Medical Center (the Netherlands) in collaboration with Freya, the Dutch association for people with fertility problems (https://www.freya.nl). The Standards for Reporting Qualitative Research (SRQR) checklist^38^ and consolidated criteria for reporting qualitative research (COREQ, see Supplemental Material)^39^ were used for reporting.

### Study population

A purposive convenience sampling strategy was used to identify Dutch-speaking women aged 18 years or older with pregnancy and/or delivery after OD in their medical history (time limit of 10 years), either with OD treatment in a Dutch or foreign centre, regardless of having a (male) partner or not. Participants were recruited by Freya via an online advertisement that was placed on their website. Women with known sex-chromosomal abnormality, or with pregnancies with foetal anomalies were excluded, as they might have received different counselling and healthcare during pregnancy. The partners of all participating women were also approached for participation in a separate focus group to explore possible differences between men and women. One focus group included a minimum of five and a maximum of eight participants in order to stimulate group dynamics and discussion.^40^ Participant recruitment and data collection lasted from September 2021 to June 2022. The study protocol was approved by the Medical Ethics Committee of the Leiden University Medical Center (N22.024). Written informed consent of all participants was obtained for audio recording, also reassuring confidentiality and anonymity in data processing and reporting. All methods were carried out in accordance with relevant guidelines and regulations.

### Data collection

The focus groups started with an introductory round to obtain socio-demographical data from the participants, and some general data on OD treatment (e.g. location, indication for OD, donor), pregnancy and delivery (e.g. complications, type of care, mode of delivery). Afterwards, each focus group was structured in three to four themes (Preconception, Pregnancy, Delivery and Organization of Care/Guideline Development), predefined by the involved researchers based on practice and to maintain a logical sequence of experiences. For each theme, semi-structured questionnaires were used to start and guide the discussion (see Supplemental Material: Questionnaire). In the first focus group, the theme ‘Delivery’ overlapped in experiences with the theme ‘Pregnancy’ and was therefore merged in the next focus groups. All focus groups were online due to the COVID-19 pandemic with an active contribution of all participants. The external contacts officer of Freya (M.V., MSc (female)), who holds professional experience as a midwife and health scientist, chaired all focus groups. An obstetrician, maternal-foetal-medicine specialist (M.H., MD, PhD (female)) and physician researcher (K.B., MD (female)) attended the focus groups for assistance in time management and recording without interference in the discussion. Each theme discussion ended with the opportunity for all participants to add or reiterate their key points on an interactive online jam board (Google), and the data were also included in the analysis. The audio was recorded and stored until verbatim transcripts were finalized.

### Data analysis

**Figure 1.**
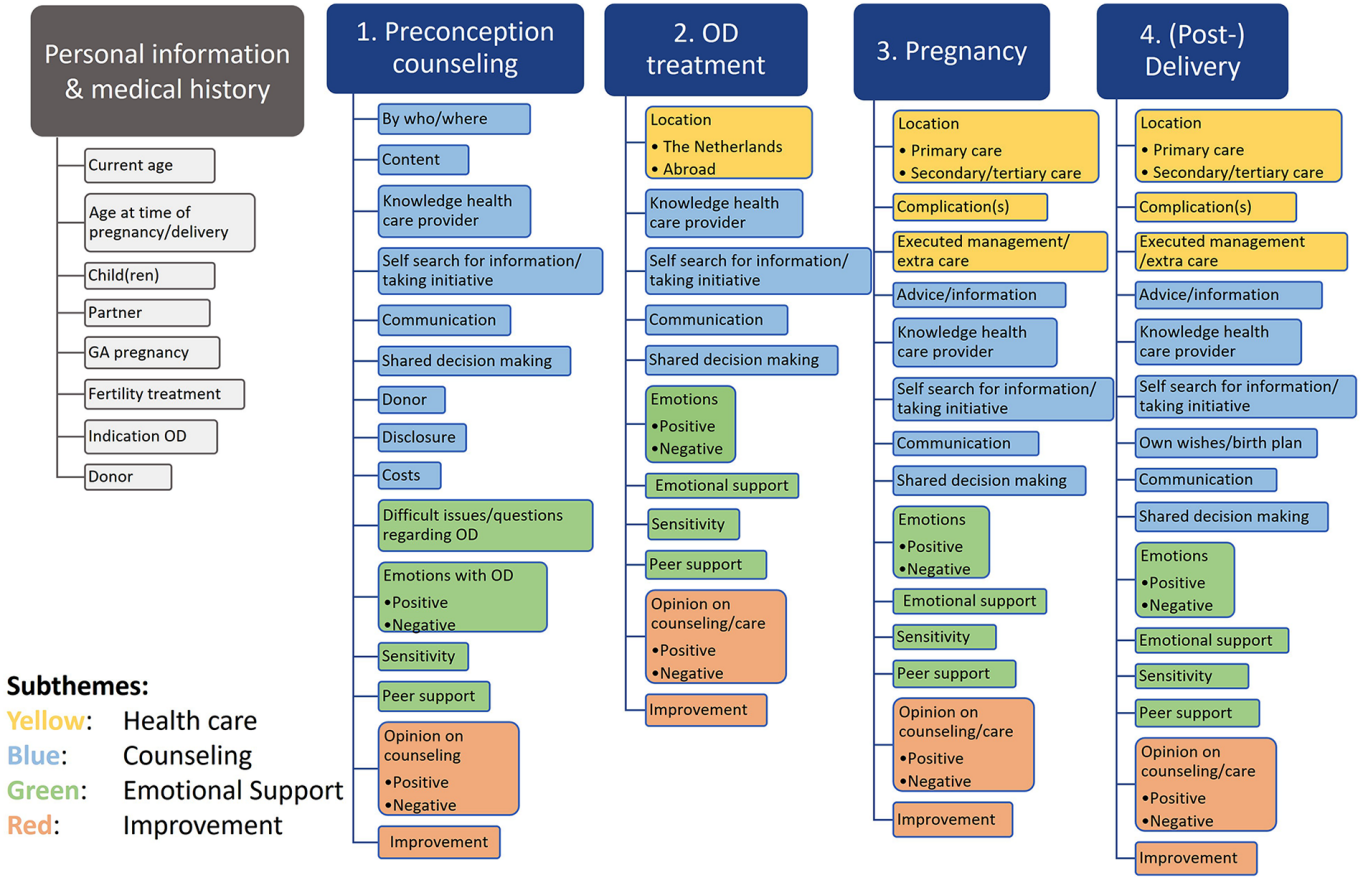
The codebook containing all 66 codes used to analyse the main themes ‘Preconception counselling’, ‘OD (oocyte donation) treatment’, ‘Pregnancy’ and ‘(Post-) Delivery’, subdivided in the subthemes ‘Healthcare’, ‘Counselling’, ‘Emotional Support’ and ‘Improvement’. The eight codes below ‘Personal information & medical history’ were used to describe participant characteristics.

All transcripts and jam board key points were imported into ATLAS.ti (©2022 ATLAS.ti Scientific Software Development GmbH, Berlin, Germany), a qualitative data analysis programme. Data were analysed with a thematic descriptive analysis approach using deductive and inductive coding.^41,42^ As the main themes of the focus groups were taken into account during the coding process, coding was partly deductive. Firstly, open coding was independently and mainly inductively assigned by two researchers (K.B. and A.V.) for focus group 1. Afterwards, the results were compared and discussed, and codes were modified and merged together when they had the same meaning. This was a reiterative process resulting in multiple cycles of coding, constant comparison and discussion by these two researchers, also taking the data of focus groups 2 and 3 into account. Once an initial set of codes emerged, the researchers conducted axial coding by organizing the data subthemes. The results of the thematic analysis were discussed with other researchers (M.H., E.L., M.K.) to reach an overall consensus of main themes, subthemes and codes. Overall, the analysis process resulted in four main themes, four subthemes and a codebook with 66 codes (see Figure 1).

## Results

### Participants

Fifteen women were willing to participate and registered through Freya, two had cancelled in advance because the scheduled time was not convenient. Focus group 1 consisted of five and focus group 2 of eight women. All women had male partners; eight of whom were willing to participate, and five who were eventually able to attend in focus group 3 at the scheduled time. None of the couples required sperm donation. The average duration of a focus group was 1 h and 42 min (group 1 2:04 h; group 2 1:32 h; group 3 1:30 h). Participant characteristics were collected in the first part of the focus group in which the participants introduced themselves and show a variety among the participants regarding location of OD treatment, (non-)anonymous donation, pregnancy complications and guidance in primary or secondary obstetric care (see Table 1).

**Table 1.** Participant characteristics of women and their male partners separately.

	Female participants				Male participants	
	FG 1 (*n* = 5)		FG 2 (*n* = 8)		FG 3 (*n* = 5)	
Variable	*N*	%	*N*	%	*N*	%
Age at focus group						
30–35	1	20	2	25	1	20
36–40	3	60	5	62.5	4	80
40+	1	20	1	12.5	0	0
Age at OD pregnancy						
30–35	3	60	3	37.5	2	40
36–40	2	40	5	62.5	3	60
40+	0	0	0	0	0	0
Children through OD						
Pregnant with first child	1	20	2	25	0	0
Pregnant with second child	1	20	1	12.5	1	20
One child	2	40	4	50	4	80
Two children	1^a^	20	1	12.5	0	0
Indication of OD						
Failed IVF/ICSI	5	100	5	62.5	3	60
POI	0	0	3	37.5	2	40
Location OD treatment						
The Netherlands	3	60	4	50	2	40
Abroad	2	40	4	50	3	60
Donor						
Anonymous	3	60	4	50	2	40
Identification by child at later age	3		3		2	
Non-anonymous	2	40	4	50	3	60
Pregnancy/delivery complications	3	60	2^b^	25	3^b^	60
PIH	1	20	1	12.5	2	40
HELLP syndrome	1	20	1^b^	12.5	1^b^	20
Gestational diabetes	1	20	0	0	0	0
PPH	0	0	1^b^	12.5	1^b^	20
Mode of delivery						
Vaginally	4	80	8	100	5	100
Caesarean section	1	20	0	0	0	0
Induction of labour	1	20	3	37.5		
Due to PIH	1	20	1	12.5	2	40
Due to OD	0	0	2	25	0	0
Pregnancy/delivery care						
Primary care	1	20	4	50	3	60
Secondary care	3	60	1	12.5	1	20
Combination	1	20	3	37.5	1	20

FG: focus group; *N*: number; OD: oocyte donation; IVF: in vitro fertilization; ICSI: intracytoplasmic sperm injection; POI: premature ovarian insufficiency; PIH: pregnancy-induced hypertension; HELLP: haemolysis, elevated liver enzymes and low platelets; PPH: postpartum haemorrhage.

a Twin.

b One participant, of whom the partner also participated, developed both HELLP syndrome and PPH.

### Thematic analysis

For each theme, the emerged data are summarized according to the subthemes and enforced with references to anonymous quotes from the transcripts that are shown in Tables 2–5.

**Table 2.** Participant quotes of ‘Theme 1: Preconception Counselling’ divided into subthemes (bold) and the corresponding codes (italics).

	**Counselling**
1	*Content* ‘Both parties [physicians from different fertility clinics] did not say anything about that [chances of ongoing pregnancy and possible pregnancy complications]’.
2	‘A higher risk for miscarriage was mentioned, but I have never been able to find confirmation in any research, so I still do not know if that is true’.
3	*Donor* ‘That [the choice for anonymous or non-anonymous donation] is very personal of course, and I was not guided in that at all. We were only asked if we had ever thought about it, but then you have to know where to start; what organizations to approach, where to go with your questions? Then you are let go and you do your own research of course’.
4	*Disclosure* ‘Everyone is different: I prefer to talk about oocyte donation all day long, to whoever wants to hear about it so to speak. However, there are women who have a different attitude’.
	**Emotions**
5	*Positive emotions* ‘In our minds, we had already abandoned the thought of ever having a child. When we heard about the existence of OD, we both found it amazing, that it is possible at all’.
6	*Negative emotions* ‘I noticed that, when we were heading towards the OD process, I really could not handle being in a Facebook group. I did not want to be involved with other women, I did not have room for that in my head. It made me so insecure’.
	**Improvement**
7	*Positive opinion* ‘Social work provided conversations about that [interaction with the donor] right away, together and separately. There was a protocol to talk about certain expectations, about what the whole process was going to look like, to see if you are suitable, so to speak. All to avoid friction between the expectation of the donor and parents. [. . .] I was very happy with it’.
8	*Negative opinion* ‘During the process, I indeed missed that the physician could not properly explain the various possibilities of oocyte donation [. . .] as far as all the countries around us are concerned; what do they offer, anonymous or non-anonymous donation? There is really no discussion of this at all’.
9	*Improvement* ‘What I have been missing is actually just people who were in the same situation as me. I felt alone. It is a very intense process, [. . .] and I had the feeling that nobody goes through this, that it was something very special. There was no help from the hospital’.
10	‘I think it might be interesting to look at a centre, because I can imagine that it [OD] is not very common, so not every physician specialises in this. It is something specialised though, so I think it would be nice to be able to go to a specialised centre where there is proper guidance’.

OD: oocyte donation.

**Table 3.** Participant quotes of ‘Theme 2: Oocyte Donation (OD) Treatment’ divided into subthemes (bold) and the corresponding codes (italics).

	**Counselling**
1	*Self-search for information/taking initiative* ‘I noticed that, especially since we went abroad, not all hospitals in the Netherlands were willing to cooperate in certain examinations with my wife. We had to go through a lot of trouble to, for example, get an ultrasound in preparation for a [embryo] transfer. I found that quite annoying, there was no guidance’.
	**Emotions**
2	*Negative emotions* ‘The preliminary process was the toughest period of my life. You assume that it [OD] is probably not going to work out, so you are also in a process of grief’.
3	‘Oocyte donation has many different aspects. Of course, the whole medical part, but what I also found very difficult was what it did in my head: not being able to deliver the oocyte yourself. What does that to me, my body, and my relationship? It has so much impact’.
4	‘When [sperm] donation makes the news, it is always bad. [. . .] Oocyte donation is more unknown. [. . .] Sometimes you read stories about the positive experiences of a family, but the news is often negative. Similarly, in opinion papers about adoption and the importance of a genetic relation between child and parent. Those things hurt sometimes, while it [OD] is such a beautiful thing for yourself and those who help you’.
	**Improvement**
5	*Positive opinion* ‘I have always found that [OD treatment counselling and care] very pleasant. We could always ask questions and also, well, the whole oocyte donation process, the whole set-up and the donor and everything, I only have good experiences. I always had the feeling that I was well informed’.
6	*Negative opinion* ‘I have not been offered social work at all, it was purely [embryo] transfers there [Spain]. You were treated like any other person, while for me it had a certain impact’.
7	*Improvement* ‘What I personally would like to see different is that the whole process of oocyte donation abroad, that it is clear how it goes, and that you are helped. At one point we were quite worried, because we got pregnant abroad, whether we would be helped in the process afterwards. And that is just something you do not want. [. . .] It is all possible, but you have to find out damn well where to go, and whether you can be helped’.

OD: oocyte donation.

**Table 4.** Participant quotes of ‘Theme 3: Pregnancy’ divided into subthemes (bold) and the corresponding codes (italics).

	**Healthcare**
1	*Executed management/extra care* ‘When I was pregnant I was quite insecure in the beginning, because you have been waiting for such a long time and know so many things that could go wrong. You do not take anything for granted anymore. Fortunately, they [obstetric care providers] were very sympathetic, so I could have extra checks. There was a lot of customization possible’.
	**Counselling**
2	*Advice/information* ‘There is a lack in contact between physicians. The fertility clinic said that I had to see a gynaecologist, while the hospital said it was not necessary in my case. Then you wonder, why do the fertility physicians and gynaecologist not have any contact about that with each other?’
3	*Knowledge healthcare provider* ‘That is something I experienced in the hospital, that of course you have numerous gynaecologists, but every gynaecologist asked us: oocyte donation, oh, but how? It was as if we were something very special’.
4	‘They [obstetric care providers] were not able to come up with research or arguments why certain management was needed. So we kept asking if there was anyone who did know the answer, but apparently nobody knew the answers to some questions’.
	**Emotions**
5	*Negative emotions* ‘Every next step [in fertility treatment] went wrong. Then you finally get pregnant and you think, okay, where can it go wrong now, it will probably end again. So, at a certain point you also become a bit miserable, you do not believe it, and you are not yet daring to be happy. You want to be standard as quickly as possible, but you also want to have extra care at the moment when you lose faith that it will go well’.
6	‘I was really happy just to be among normal pregnant women. The only thing I thought at the time was, are they [midwives] not overlooking something. Because in the preliminary process at the different hospitals, we had to come up with a lot of thing ourselves to actually get further with treatments’.
7	*Sensitivity* ‘At the ultrasound it turned out that she [foetus] had long legs. The midwife then told me that I also had long legs. Well, nice, but that was totally irrelevant’.
8	*Emotional support* ‘We came out of a very emotional rollercoaster, and I missed someone who just occasionally showed support. In that sense, it seemed that it did not matter whether I had become pregnant by accident for the fifteenth time. That feeling of all the weight you carry, I missed that’.
	**Improvement**
9	*Positive opinion* ‘When the gynaecologist said that we could go to a midwife after about five months, my girlfriend was very happy, because then she felt like she was just like everybody else. Not medical anymore, just normally pregnant’.
10	*Improvement* ‘She [gynaecologist] had no idea that this was an oocyte donation pregnancy. I found that quite notable, since that was the reason we were in the hospital’.
11	‘What I just found really crazy is that every hospital does something different with a woman who got pregnant through oocyte donation. I think that is kind of crazy to hear afterwards’.
12	‘Then I also wonder whether it is possible to ensure with a guideline that somehow all information is much better available, so that it is much clearer what has and has not been scientifically established. What are risks and what are not, and are there things we can just be more relaxed about? I think that would be very important to work on’.
13	‘It [OD process] is different for everyone, it is so specific, no story is the same. [. . .] But it is good to ask all couples what their needs [for emotional support] are’.

OD: oocyte donation.

**Table 5.** Participant quotes of ‘Theme 4: (Post-)Delivery’ divided into subthemes (bold) and the corresponding codes (italics).

	**Counselling**
1	*Knowledge healthcare provider* ‘That [risk for retained placenta] was something that caused a sort of commotion in the [Facebook] group, while a lot of gynaecologists did not really know whether or not that was a risk. [. . .] Yet another example of information that is present, but is it correct and from who is it coming?’
2	‘I was told from the beginning that my pregnancy was not going to pass 40 weeks, because of oocyte donation. When I tried to ask why not, what are the risks, I got the answer “we do not really know, but that is in our guideline”’.
3	*Communication* ‘Then you are dismissed from the hospital and you get care from a midwife you have never seen before for the follow-up at home, who asks you about contraception. Then I think, seriously, well that kind of stupid things, I just do not understand that’.
	**Emotions**
4	*Negative emotions* ‘Breast feeding was a big drama for me, I felt a bit pressured, or put under pressure [. . .]. But because of the oocyte donation, I guess I already had feelings of failure, so obviously I could not do that [provide own oocytes] and I probably could not do that [breastfeed] too’.
5	‘The moment my son was there, [. . .] I was very anxious about whether that feeling would be there. Like this is my baby, even though you know it is an oocyte donation baby’.
6	*Sensitivity* ‘I always appointed to all the gynaecologists that it was an oocyte donation pregnancy, and later with the delivery I really wanted everyone at my bedside to know that. That they do not immediately start saying “that baby looks exactly like its mother”. I was really afraid of that, and so I had to keep pointing that out. [. . .] That sensitivity is something we encountered’.
	**Improvement**
7	*Positive opinion* ‘In my maternity bed, the midwife came by [. . .], and she asked me: “How do you feel about the oocyte donation? [. . .] Do you recognise your child as yours, and how does your donor look at it?” It was actually very pleasant to be able to talk about it, it makes you feel seen’.
8	*Negative opinion* ‘Physicians differ within one hospital: where one gynaecologist reacts a bit alarmed about it [OD], the other gynaecologist allows to give birth at home. [. . .] How are you supposed to filter what is really important and who is right? Because they are the ones who have been educated for this, and are supposed to say what is true and what is right’.
9	*Improvement* ‘It would also help a lot if there was a guideline. Look, there will always be differences between what you want as a pregnant woman [regarding delivery] and what the physician wants, but yes, having something on paper would be very helpful’.

OD: oocyte donation.

### Theme 1: preconception counselling

‘Both parties [physicians from different fertility clinics] did not say anything about that [chances of ongoing pregnancy and possible pregnancy complications]’.

#### Counselling

Participants received preconception counselling by various healthcare providers (fertility physicians, gynaecologists, general practitioners, midwives, psychologists and social workers), in different forms (verbal, (online) folders and books). Fertility physicians and gynaecologists were mentioned as counsellors on the medical stages of the OD process and possible (pregnancy) complications. Apart from one couple, all participants who had received OD treatment in the Netherlands had been informed about possible pregnancy complications during preconception counselling (Table 2; quote 1). Of the nine participants (including two couples) who went abroad for OD treatment, six (including one couple) were preconceptionally counselled about pregnancy risks by their fertility physician. Participants appreciated to have an open conversation, the possibility to ask questions and shared decision-making. However, some expressed scepticism regarding the reliability of the information from fertility physicians, as they felt a lack in knowledge about OD and received ambivalent information and advice. As a result, participants had to take initiative to obtain information and searched for example through peer Facebook pages and online forums (Table 2; quote 2). All participants, except for one, who received OD treatment in the Netherlands were consulted by a psychologist or social worker for preconception counselling on the psychosocial and ethical part of OD, and all expressed positive experiences (Table 2; quote 7). Not all couples were able to find a suitable donor themselves or through the donor bank and were left with the only other option to go abroad. The participants who went to Finland were obliged to receive counselling from a social worker in the Netherlands, which was lacking for other foreign clinics. Some participants reported that they would prefer to receive better counselling about the challenge to find a suitable anonymous or non-anonymous donor abroad (Table 2; quote 3), which introduces the issue about the disclosure of OD (Table 2; quote 4).

#### Emotional support

The news that OD was the only option left to conceive caused different emotional reactions among the participants (Table 2; quote 5). Some participants mentioned that the preconception counselling on OD came too soon, as they still had to process the bad news from the previous fertility programme. Furthermore, participants expressed negative emotions towards Dutch fertility care providers who were not allowed to give information about OD treatment abroad because of opposite legislation and ethical constraints. Emotional support was valued among all participants, and peer support was a frequently recurring topic during the focus groups. Some participants who did not receive help from fertility care providers in where to find peer support reported feelings of loneliness and insecurity, while others were reluctant in finding peer support so early in the process (Table 2; quote 6).

#### Improvement

To make preconception counselling more accessible, it was suggested that all readable forms, such as a folder and website, should be consistent and updated with the latest findings from research. Furthermore, emotional and peer support were valued by some even before conception, though the help from fertility care providers in finding this was raised as point of improvement (Table 2; quote 9). Most importantly, participants noticed ambivalence in advice and felt a lack of knowledge on OD among fertility care providers. In addition, they missed the possibility to get information about OD treatment abroad from fertility care providers in the Netherlands (Table 2; quote 8). Participants recommended to spread uniform knowledge to accommodate fertility care providers and improve preconception counselling, for example through a national guideline, scientific journals or a specialized centre (Table 2; quote 10).

### Theme 2: OD treatment

‘Oocyte donation has many different aspects. Of course, the whole medical part, but what I also found very difficult was what it did in my head: not being able to deliver the oocyte yourself. What does that to me, my body, and my relationship? It has so much impact’.

#### Healthcare

Participants were treated in the Netherlands or abroad, resulting in different perspectives about OD treatment care, and about both known and anonymous donors. The couples who went abroad for OD faced difficulties in the collaboration with Dutch fertility care, for example in the cooperation with certain examinations necessary for embryo transfer (Table 3; quote 1). Participants who had OD treatment in the Netherlands also faced some problems in the cooperation and logistical knowledge of fertility care providers but were generally satisfied with the obtained healthcare during OD treatment (Table 3; quote 5).

#### Counselling

With evident preconception counselling, the participants were able to make an autonomous choice to start OD treatment. Participants appreciated to receive a clear explanation about their fertility problems, even if it was bad news, and the upcoming OD treatment. However, lack of information by fertility physicians sometimes resulted in having to find information on own initiative, for example about their fertility problems. Abroad, counselling during OD treatment was mostly focused on the medical stages of the treatment, and some participants were already counselled on certain policies during pregnancy by their foreign fertility physician.

#### Emotional support

Positive emotions during OD treatment were evoked by gratitude towards the donor, and quick success of the OD treatment. However, the process of OD was unanimously described as physically and mentally demanding by both the women and their partners (Table 3; quote 2). Firstly, the women needed to accept the fact that they were not able to conceive with their own oocytes (Table 3; quote 3). In addition, the partners revealed male-specific factors, as worries about sperm quality were not always well addressed due to the focus on the oocyte problems. Also, negative opinions about gamete donation from outsiders, for example in the national news, had impact (Table 3; quote 4). No negative emotions were shared about the opinion from family and friends, who actually provided more support. Fertility care providers also helped with emotional support during OD treatment by showing interest and empathy. However, continuity of care by a single physician was often not present, which made it more difficult to receive emotional support from a confidential fertility physician. The relevance of peer contact and the possibility to consult social work or a psychologist for emotional support was again emphasized by the participating women. However, these consultations were missed by some participants who went abroad for OD treatment (Table 3; quote 6).

#### Improvement

The most stated point for improvement by participants who went abroad for OD treatment was the lack in cooperation of Dutch fertility care providers (Table 3; quote 1 and 7). In line with what was previously mentioned as improvement for preconception counselling, the participants again recommended to promote dissemination of knowledge among fertility physicians, for example through conferences and a national guideline. Likewise, more guidance from fertility physicians in seeking emotional support from a professional was cited.

### Theme 3: pregnancy

‘What I just found really crazy is that every hospital does something different with a woman who got pregnant through oocyte donation. I think that is kind of crazy to hear afterwards’.

#### Healthcare

The participants experienced varying complications and obstetric care management during OD pregnancy. Both primary midwifery care and secondary hospital care were provided, frequently accommodated with somewhat personalized OD pregnancy care, such as extra ultrasounds or prescription of aspirin (Table 4; quote 1). The need for secondary care was not always clear and reasoning for it differed among obstetric care providers (Table 4; quote 2). Some participants appreciated that OD was seen as an indication for secondary care; however, notable were multiple statements about being seen as a normal pregnant woman in primary care (Table 4; quote 9). Most participants were aware why additional management in obstetric care was needed because of OD, though many differences emerged (Table 4; quote 2). A few participants were unaware and heard certain management options for the first time during the focus group, indicating that these were not known or implemented by the obstetric care provider.

#### Counselling

Counselling during OD pregnancy might come with changing management, for example when complications arise. Whenever a change in policy occurs, straightforward communication and shared decision making are important. However, the knowledge of obstetric care providers about OD was questioned, counteracting appropriate counselling during OD pregnancy (Table 4; quote 3). It was mentioned multiple times that physicians and midwives were unable to support their advice and policy with evidence-based studies and arguments, or to refer their patients to a fellow healthcare provider who had the required knowledge (Table 4; quote 4).

#### Emotional support

Finally achieving pregnancy brought new emotions for the participants, mostly positive. These positive emotions were enforced when pregnancy went well, management and communication were clear and empathy for the long process of conceiving was shown, thereby also taking the feelings of the partner into account. Nevertheless, participants also experienced negative emotions, partly due to their long fertility treatments in the past, that led to feelings of uncertainty and sometimes distrust in the obstetric care provider (Table 4; quotes 5 and 6). In addition, negative emotions were related to the absence of sensitivity for OD from obstetric care providers (Table 4; quote 7). Lastly, follow-up of emotional support during pregnancy was often desired, but not always offered (Table 4; quote 8).

#### Improvement

For the provision of the right OD pregnancy care, a proper data transfer between fertility and obstetric care providers is needed, though often highlighted as a point for improvement (Table 4; quote 10). The participants sense a lack in knowledge on OD among obstetric care providers, resulting in ambivalent advice and management of OD pregnancy care, and confusion among participants (Table 4; quotes 11). Again, the participants emphasized the need for reliable information, for example by implementing a guideline to have an outline for healthcare management during OD pregnancy (Table 4; quote 12). Furthermore, being sensitive about OD, offering emotional support and informing about peer support should be taken into account by obstetric care providers when encountering a couple that conceived through OD (Table 4; quote 13).

### Theme 4: (post-)delivery

‘It would also help a lot if there was a guideline. Look, there will always be differences between what you want as a pregnant woman [regarding delivery] and what the physician wants, but yes, having something on paper would be very helpful’.

#### Healthcare

Again, the participants discovered various differences in executed policies around delivery. While some were required to deliver in the hospital with secondary care, others still had the choice to deliver at home or in the hospital under guidance of primary care (Table 5; quote 8). Moreover, differences emerged regarding induction of labour; some hospitals provided induction as standard care after OD, while others did not see the need for it if no pregnancy complications occurred.

#### Counselling

Participants wanted to be counselled about their options for the location of delivery and possible risks that could occur during delivery because of OD, for example the risk for retained placenta. However, they noticed a lot of ambiguity in the information supply (Table 5; quote 1). Not all participants were content with the counselling they received due to absence of good argumentation, for example about induction of labour, and lack of shared decision-making (Table 5; quote 2). In parallel, other participants had experienced good counselling, and they were able to give input on delivery management. Regarding the postpartum period, some remarks were made about possible troubles with breastfeeding after OD, as participants received contradictory information from obstetric care providers. Furthermore, the importance of a complete data transfer between obstetric care providers after delivery was discussed, as the participants faced difficulties due to lacking communication about OD (Table 5; quote 3).

#### Emotional support

Delivery was referred to as an emotional outlet of all that had happened in the past. Although some women did put a lot of pressure on themselves to make the delivery and postpartum period succeed and not experience failure again (Table 5; quote 4), others had a more relaxed and positive mindset. Some participants mentioned that they experienced distress when thinking about the bonding process with their new born (Table 5; quote 5). Obstetric care providers who showed empathy and interest in issues and emotions related to OD were positively evaluated (Table 5; quote 7), while some participants mentioned that sensitivity for OD was missing during delivery and postpartum (Table 5; quote 6).

#### Improvement

Regarding (post-)delivery care and counselling, the participants reported that unambiguous counselling on management during delivery after OD pregnancy is very important, as many differences between obstetric care providers were noticed. Possible solutions that were mentioned mainly involved better supply of information, shared decision-making and the development of a guideline (Table 5; quote 9). Correspondingly, the need for better guidance in emotional and peer support after OD pregnancy was stated. Lastly, flaws in the communication between obstetric care providers and lack of sensitivity for OD were addressed, for which adequate filing and transfer of patient data were cited as points for improvement.

## Discussion

This exploratory qualitative study explores the perspectives of oocyte recipients and their partners on OD counselling and healthcare and presents their needs and recommendations for improvement. By organizing focus group data into four themes, topics of satisfaction and improvement regarding counselling, healthcare and emotional support were summarized for each stage of the OD process. Although OD (preconception) counselling and care raised various positive experiences, such as consultations for emotional and ethical support before conception and personalized OD pregnancy care, other perspectives resulted in points for improvement. The perspectives that emerged most prominently included the feeling of a lack in knowledge about OD among healthcare providers, contradictory information and advice in counselling, lack in consensus on OD pregnancy and delivery care management and the need for emotional and peer support. Psychosocial support before the start of OD treatment is offered in the Netherlands, but often absent when treated abroad. Furthermore, follow-up of emotional support during and after pregnancy is often desired, but not always provided. Therefore, various recommendations for the improvement of OD healthcare management were stated by the participants; mostly appointed was the implementation of a (inter)national guideline.

The key point that emerged from our focus group data is the importance of adequate counselling, accounting for a significant part of guidance during the entire OD process. Preconception counselling was positively evaluated when various subjects were covered, not only the logistical part of OD, also the emotional impact and ethical issues of OD. The importance of preconception counselling before OD treatment was already acknowledged by previous research.^43–45^ As these studies have been done several years ago, our focus group data demonstrate that preconception counselling is considered more often. Still, the data suggest that certain subjects are not adequately addressed during counselling, such as the higher risk for pregnancy complications. A recent qualitative interview study explored OD recipients’ understanding of the risks associated with OD pregnancy and, consistent with our findings, found that most were unaware of the higher risk profile compared to in vitro fertilization (IVF) pregnancy. Three key themes emerged: women focused mainly on treatment success, lacked knowledge of specific risks and showed ambivalence towards receiving risk information. Corresponding to our results, this study underscores the importance of enhancing risk counselling during the consent process and routinely assessing women’s understanding during antenatal care.^46^ Furthermore, lack in knowledge and conflicting information and advice from OD healthcare providers were recurring points of discussion in every part of the focus groups. Nowadays, more and more research has been done on OD pregnancy complications,^7–10^ also proposing options to decrease complication risks.^6^ As participants’ perspectives show that consensus on the management of OD pregnancy and delivery is missing among healthcare providers, these results could contribute to the development of a guideline on healthcare management related to OD.

Emotional support throughout the OD process was broadly discussed and mostly appreciated by the participants. When preparing for OD treatment in the Netherlands, consultation with a social worker or psychologist is standard of care, and likewise, it is strongly advised by the American Society of Reproductive Medicine.^47^ Psychological counselling is often needed for OD recipients and their partners to reflect on the use of OD, explore its emotional impact and recognize the need for therapy to address inner conflict and distress.^48^ Our study showed that a variety of feelings can arise among OD recipients and their partners, which is in line with earlier studies.^11,49^ Ghelich-Khani et al.^11^ found that the OD process could be followed by the experiences of distressing psychologic symptoms, social stigmatization and negative coping mechanisms in recipient women. A validated questionnaire study in the United Kingdom showed that most women from the general population had little to no knowledge about OD, but after explanation, most approved of OD (85.8%).^50^ Nevertheless, stigmas around OD persist which are often linked to cultural and religious beliefs. In an Iranian OD population, social stigmatization could be divided into three subcategories: concerns about disclosure, judgement of others and conflict with religious teachings. The OD recipients faced emotional distress from childlessness while also feeling compelled to hide the use of OD due to fear of social stigma and judgement, as was reported by 55%. Concerns included negative reactions from others, being blamed for infertility, dissimilarity of the parents with the offspring and religious conflicts with 35% initially considering OD as unethical or against Islamic teachings.^11^ Similarly, in Greece, where having a child is highly valued and motherhood is seen as essential to a woman’s identity, childlessness is stigmatized and OD viewed as morally unacceptable by the Orthodox Church.^51^ According to the Dutch Central Bureau of Statistics, the majority of the population in the Netherlands identifies as non-religious, while among those who are religious, Roman Catholicism is the most prevalent, followed by Protestantism and Islam.^52^ However, as we did not collect data on participants’ religious affiliations, we are unable to draw any conclusions regarding potential religious matters related to OD.

A Finnish study by Sälevaara et al.^32^ indicated that OD mothers had less mental health symptoms in early parenthood in comparison with women who conceived with their own oocytes. This result contradicted their hypothesis, possibly explained by intensive preparation for parenthood, the realization of an almost impossible dream and selection effects of partnerships that are strong enough to withstand stress caused by the OD process. In addition, a British study found no significant differences in psychological well-being or family relationship quality between assisted reproduction by gamete donation and unassisted conception families. However, within gamete donation families, earlier disclosure of biological origins (before age 7 of the child) was linked to better mother–child relationships and lower maternal anxiety and depression.^53,54^ Though parent–child interaction quality was similar between OD and IVF families, OD parents reported higher parenting stress, lower confidence and more negative emotions towards their children.^55^ To summarize, awareness of mental health and emotional support should not be forgotten by healthcare providers in all the phases of the OD process. Research showed that women who went abroad for OD treatment have higher levels of anxiety compared to local women.^56^ Additional attention should be given to the possibility that OD recipients missed emotional support, which also emerged in the focus groups.

Another notable finding was the often valued support from peers, though couples experienced lack in guidance how to get in touch with peers. In a donor insemination (semen) programme, it was found that peer support decreases feelings of isolation and stigma by normalizing donor experiences. In addition, peer support offered new information resulting from the shared personal experiences from couples in the same situation.^57^ It should be considered, however, that the current study population may have been biased regarding the need for peer support, as they were recruited through a patient association.

The issues of finding a suitable donor and counselling about OD treatment abroad were emphasized by the participants. Dutch healthcare providers are not allowed to counsel on OD abroad due to legislation, as commercial and anonymous donation is forbidden by law.^58^ In some countries, the legislation resembles that of the Netherlands; the child has the opportunity to request data of the donor after a certain age (e.g. Portugal, Finland, United Kingdom). Other countries only allow anonymous donation (e.g. Spain, Czech Republic, Cyprus, Russia, Italy, Greece), offer both anonymous and non-anonymous donation (e.g. Belgium, United States) or prohibit to execute OD completely (Germany, Switzerland, Norway).^4,59^ Patients will have difficulties in finding a suitable donor, but research on this topic is lacking. Though the current research was conducted with and focused on the Dutch population, it certainly showed factors in OD counselling and healthcare that are also important for policy in other countries. Since certain ethical constraints regarding OD in the Netherlands and other countries are drivers for cross-border OD treatment, this indicates the importance for each country to take steps in improving OD counselling and healthcare.

Lastly, the focus group participants mentioned various recommendations to improve OD counselling and healthcare, in which the development of a (inter)national guideline was cited most frequently. A guideline could contribute in disseminating uniform knowledge and medical management to accommodate OD healthcare providers, possibly resulting in less ambivalent advice and confusion among OD patients. Recently, more guidelines have been published that specifically address gamete donation. Both the ESHRE and ASRM developed good practice recommendations for the information provision, guidance and physical and psychological evaluation of involved stakeholders in gamete donation.^47,60^ These recommendations are a stepping stone towards (inter)national guidelines and could be used for a (Dutch) population specific, evidence- and practice-based, summarizing, and easy-to-find guideline in order to improve OD healthcare management. Various good practice recommendations of the ESHRE are in line with our study, as it is recommended to signpost recipients to available sources (e.g. books, websites, peer support) to inform recipients about the different forms of donation, and medical risks.^60^ While the current study demonstrates that there are still improvements for clinical practice to achieve regarding OD counselling, it also shows that various patient perspectives do correspond with the view of professionals.

### Limitations

This is the first study that clarified the perspectives of women and their partners regarding both counselling and healthcare before and during OD pregnancy in order to improve OD healthcare management. The mostly inductive thematic analysis identified themes, subthemes and codes that are strongly linked to the data. However, it is often not possible for researchers to completely release their own perspectives and assumptions in the highly interpretive analytic process.^38^ Nevertheless, the investigators showed reflexivity by being aware of their own positionality, assumptions and beliefs throughout the research process.^61^ By independently coding the data by multiple researchers, the validity of the results was increased. In addition, data triangulation occurred by collecting data from different individuals belonging to different groups, namely women and their male partners, receiving OD treatment in the Netherlands or abroad, in order to obtain multiple perspectives and validation of data. Furthermore, multiple investigators corroborated on the findings, enhancing investigator triangulation and credibility of the results.^61,62^ However, it cannot be definitively stated that data saturation was achieved after the third focus group, as new information might have emerged with additional focus groups.^61,63^

The form of participant recruitment could introduce selection bias, as participants had to register themselves and only women who were active on the Freya website were exposed to the advertisement. This selection possibly caused underreporting of maternal health screening before start of OD treatment because no negative experiences occurred among the participants, since all were allowed to start OD treatment. Furthermore, the experiences of infertility and OD in couples could differ culturally, religiously and legally from other couples, and therefore, possible selection bias is important to note. Furthermore, all participating partners were male, which does not represent the emerging trend of shared lesbian motherhood.

Another limitation is the possibility of recall bias, as OD treatment, pregnancy and/or delivery had been a while for some participants. This was managed by restricting inclusion with a 10-year time limit after OD pregnancy. In addition, the attendance of a medical specialist in the focus groups could have hindered patients in sharing experiences, though no patient-physician relationship was present. In future research, the number of and diversity in participants could be increased by also recruiting via, for example, fertility clinics or hospitals. Furthermore, to reduce recall bias, this research can be designed prospectively by planning evaluation moments during the OD process.

## Conclusion

This qualitative study showed various perspectives on OD counselling and healthcare of different women and their partners and also addressed associated emotional concerns, in order to improve OD healthcare management. Considering the possible medical, emotional and ethical risks of OD, it is of utmost importance to provide evidence- and experience-based, and comprehensive (preconception) counselling. As these women will not have an alternative method to conceive, healthcare providers should be aware of the risk profile and offer proper counselling and (emotional) healthcare throughout OD treatment, pregnancy and (post-) delivery. The results of this study will facilitate the development of a (inter)national guideline, that offers guidance to OD healthcare providers. Currently, such a guideline, including advice on counselling, healthcare policy and emotional support for women during their OD pregnancy, is non-existing. In this regard, it is also important to identify the perspectives of other involved stakeholders in future research, such as fertility specialists, obstetricians and oocyte donors.

## Supplemental Material

sj-docx-1-whe-10.1177_17455057251374891 – Supplemental material for The perspectives of recipients and their partners conceiving through oocyte donation on counselling and healthcare: A qualitative study Supplemental material, sj-docx-1-whe-10.1177_17455057251374891 for The perspectives of recipients and their partners conceiving through oocyte donation on counselling and healthcare: A qualitative study by Kim van Bentem, Eileen Lashley, Amber Visser, Marloes Vermeulen, Moniek ter Kuile and Marie-Louise van der Hoorn in Women’s Health

sj-docx-2-whe-10.1177_17455057251374891 – Supplemental material for The perspectives of recipients and their partners conceiving through oocyte donation on counselling and healthcare: A qualitative study Supplemental material, sj-docx-2-whe-10.1177_17455057251374891 for The perspectives of recipients and their partners conceiving through oocyte donation on counselling and healthcare: A qualitative study by Kim van Bentem, Eileen Lashley, Amber Visser, Marloes Vermeulen, Moniek ter Kuile and Marie-Louise van der Hoorn in Women’s Health
