# Supplementary material for: The perspectives of recipients and their partners conceiving through oocyte donation on counselling and healthcare: A qualitative study
Source: Womens Health (Lond). 2025 Oct 7;21:17455057251374891. doi: 10.1177/17455057251374891 (PMC12504846; doi:10.1177/17455057251374891)
Supplement: sj-docx-1-whe-10.1177_17455057251374891 – Supplemental material for The perspectives of recipients and their partners conceiving through oocyte donation on counselling and healthcare: A qualitative study [file sj-docx-1-whe-10.1177_17455057251374891.docx]

**Supplementary material: Questionnaire**

Focus group themes and general questions to guide the discussion.

| Themes | General questions |
| --- | --- |
| Preconception | - Was information provided about risks of an OD pregnancy (preconception counselling)?   - If so, what information and by whom was this provided?   - What did you like about this, what was less good?   - Did you miss any information (afterwards)?   - Did you search for or find information yourself? |
| Pregnancy | - Who provided your pregnancy care (midwife, gynaecologist, or combination)?   - What is your opinion about this?   - Did you have any influence in that yourself (e.g. ask for particular pregnancy care)?   - Did you receive any counselling on OD pregnancy? What information was discussed with you and what was your opinion?   - Did you receive ‘extra’ care because of OD pregnancy? If so, what ‘extra’ care (e.g. fetal growth ultrasound, aspirin, referral to gynaecologist)? If not, did you miss any care? - Would you like extra attention for the fact that you became pregnant through OD? |
| Delivery | - What was the support around the delivery like? Were you allowed to opt for home delivery? Who accompanied you? Was there continuous fetal monitoring? Was your delivery induced?   - What is your opinion?   - Did you receive 'extra' care around your delivery because of OD pregnancy? If so, what ‘extra’ care? If not, did you miss any care? |
| Organization of Care / Guideline Development | - Do involved caregivers have enough knowledge to properly counsel women who are pregnant through OD?   - What makes you think so? Is there anything you missed? Is there a difference between caregivers? - If a national guideline is developed on counselling and care related to OD pregnancy, what should be included? - How would you like to receive information/counselling on OD treatment and pregnancy (e.g. information folders, website, from a provider)? - What is your opinion on taking other factors, next to OD, into account in deciding on appropriate pregnancy care? If so, what factors (e.g. maternal age, complications in previous pregnancy, genetic relation between mother and donor)? |
